# Causal drivers of mosquito abundance in urban informal settlements

**DOI:** 10.1088/1748-9326/add751

**Published:** 2025-05-20

**Authors:** Emma E Ramsay, Peter A Faber, Genie M Fleming, Grant A Duffy, Andi Zulkifli Agussalim, S Fiona Barker, Maghfira Saifuddaolah, Ruzka R Taruc, Autiko Tela, Revoni Vamosi, Silvia Rosova Vilsoni, Steven L Chown

**Affiliations:** 1 School of Biological Sciences, Monash University, Melbourne, Victoria 3800, Australia; 2 Asian School of the Environment, Nanyang Technological University, Singapore 639798, Singapore; 3 Research & Enterprise, University of Otago, Dunedin, New Zealand; 4 RISE Program, Faculty of Public Health, Makassar, Hasanuddin University, Makassar, Indonesia; 5 School of Public Health and Preventative Medicine, Monash University, Melbourne, Victoria 3004, Australia; 6 RISE Program and School of Public Health and Primary Care, Fiji National University, Suva, Fiji

**Keywords:** informal settlements, mosquito, mosquito-borne disease, water security, sustainable development

## Abstract

Urban informal settlement residents are vulnerable to mosquito-borne diseases, but little is known about the specific drivers of risk, or how they differ, within the diversity of informal settlements globally. Here we aimed to identify key drivers of mosquito abundance in different urban informal settlements to inform upgrading programs. We developed a causal framework of mosquito risk and tested it in two distinct geographic settings: Makassar, Indonesia and Suva, Fiji. Using longitudinal mosquito trapping surveys in 24 informal settlements between 2018 and 2024 (totalling 1534 successful trap sets in Makassar and 1216 in Suva), we fitted causal models to infer the relationships between climatic, environmental and socioeconomic drivers and the abundance of two dominant mosquito species: *Aedes aegypti* and *Culex quinquefasciatus*. Water supply and access, and variation in temperature and precipitation were key drivers of mosquito abundance in both informal settlement locations, but the direction of effects differed between vector species. Piped water supply in a settlement reduced the abundance of the dengue vector, *Ae. aegypti* but increased the abundance of *Cx. quinquefasciatus.* Higher temperature and precipitation were associated with more *Ae. aegypti* in both geographic locations. By identifying the pathways through which changes in informal settlement environments are likely to alter mosquito risk we provide essential information to guide upgrading and resilience programs.

## 1. Introduction

Rapid urbanisation and population growth across the tropics have contributed to the growing burden of mosquito-borne diseases. Viruses including dengue, Zika and West Nile are increasing in incidence globally and expanding in their distributions (Franklinos *et al* 2019). Yet considerable variation in the risk of mosquito-borne diseases exists, both within and between cities (Franklinos *et al* 2019). In urban settings, climate variables, including temperature and precipitation, interact with socioeconomic and environmental factors to have contrasting effects in different communities, and on different vectors (Alirol *et al* 2011, LaDeau *et al* 2015).

Low-income communities tend to have a higher burden of mosquito-borne disease (Alirol *et al* 2011, Costa *et al* 2017, Rosser *et al* 2025). Among the most vulnerable are those living in urban informal settlements, characterised by insufficient services and infrastructure, and home to an estimated 1 billion people globally (Ezeh *et al* 2017, Satterthwaite *et al* 2020, United Nations 2023). A lack of piped water supply, as is typical in informal settlements (Ezeh *et al* 2017), promotes water storage, creating breeding habitat especially favoured by the primary dengue vector, *Aedes aegypti* (Alirol *et al* 2011). Low precipitation and drought, which exacerbate water insecurity, can amplify this effect (Lowe *et al* 2021), whilst high precipitation and flooding can alternatively fill or flush mosquito breeding habitat (e.g. in drains or hard waste; Coalson *et al* 2021). Meanwhile, urban heat islands cause considerable microclimate variation in urban environments which can have similarly variable effects on different vectors (Mordecai *et al* 2019). Informal settlements tend to have distinct thermal profiles compared to other urban areas, and the dearth of heat mitigation, including in houses, is likely to amplify any thermal effects on vectors (Ramsay *et al* 2021, Wilby *et al* 2021).

Mosquito dynamics are, therefore, likely to play out differently in urban informal settlements compared with other urban settings, and between informal settlements, given the diversity of their contexts globally (Ezeh *et al* 2017, Hambrecht *et al* 2022). Moreover, informal settlements are often the subject of upgrading programs through which changes to the biophysical environment may inadvertently or intentionally alter mosquito dynamics (Olthuis *et al* 2015). Yet the drivers of risk at local scales relevant to informing interventions, and the extent to which these are generalisable across different informal settlement contexts, are poorly understood. Multi-settlement investigations of the climatic, environmental and socioeconomic drivers of mosquito dynamics are, therefore, essential for improving human health and livelihoods (Costa *et al* 2017, Lilford *et al* 2017), in keeping with Sustainable Development Goals 3 (Good health and wellbeing) and 11 (Sustainable cities and communities; United Nations 2023).

**Figure 1.**
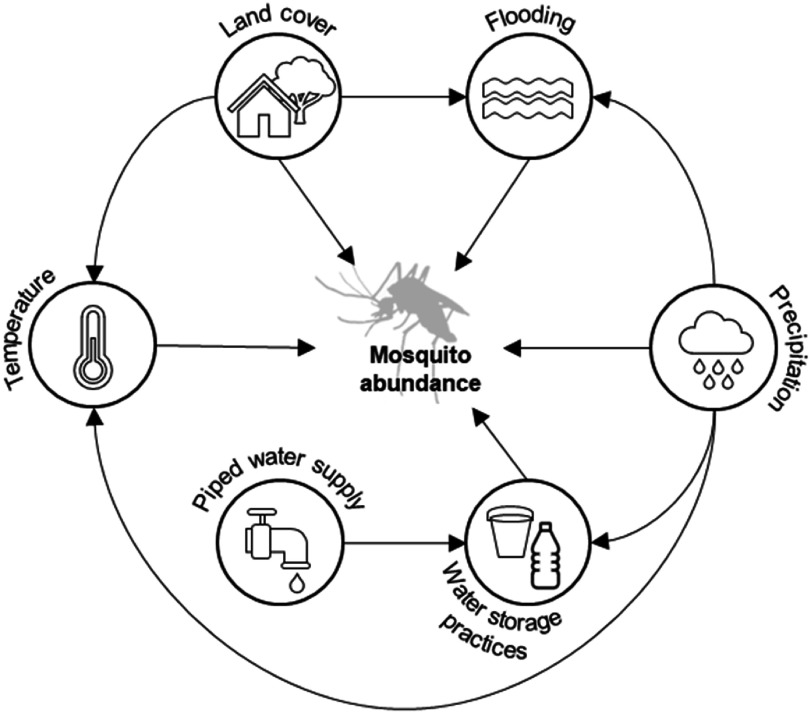
Putative climatic, environmental and socioeconomic drivers of mosquito abundance in urban informal settlements. Directed acyclic graph shows the causal relationships between climatic, environmental and socioeconomic drivers and mosquito abundance in urban informal settlements. See table S1 for the rationale for each relationship.

Here we modelled the causal drivers of mosquito dynamics in two geographically distinct urban informal settlement contexts. We developed a causal framework of the climatic, environmental and socioeconomic drivers of mosquito abundance (figure 1) and tested these relationships using longitudinal mosquito trapping data from Makassar, Indonesia and Suva, Fiji. By following a causal framework approach (Arif and MacNeil 2022) we explicitly accounted for the interdependence between climatic, environmental and socioeconomic drivers, and examined their transferability across settings, and between vector species. In doing so we aimed to identify the pathways through which interventions may intentionally or inadvertently alter mosquito dynamics and the risk of mosquito-borne disease in urban informal settlements.

## 2. Methods

Mosquito abundance, climatic, environmental and socioeconomic data were collected in 12 informal settlements in Makassar, Indonesia (5.1616° S, 119.4359° E) and 12 informal settlements in Suva, Fiji (18.1405° S, 178.4233° E) between 2018 and 2024 as part of the Revitalising Informal Settlements and their Environments (RISE) program (Leder *et al* 2021). Both countries have tropical climates yet represent distinct urban settings (Leder *et al* 2021). Makassar is a densely populated city with a population of 1.5 million people, located on the island of Sulawesi, Indonesia (French *et al* 2021). With a population of 93 000 people, Suva, located on the island of Viti Levu, Fiji is a much smaller city, characterised by low density urban development (Leder *et al* 2021). Informal settlements comprise 30% and 11% of the urban population in Indonesia and Fiji, respectively (United Nations 2021). Households in informal settlements in both countries are characterised by poor quality housing and low prevalence of air-conditioning (French *et al* 2021, Leder *et al* 2021).

### 2.1. Mosquito abundance

Adult mosquito traps were deployed quarterly or 6 monthly (following COVID-19-induced fieldwork limitations) in 15 houses in each informal settlement (from those consented for the RISE Program; Leder *et al* 2021), randomly selected in the first round of sampling. In Makassar, 12 sampling rounds were conducted between October 2018 and January 2024. In Suva, 11 sampling rounds were conducted between August 2019 and January 2024 (table 1). Longer gaps between mosquito surveys occurred in 2020 in Makassar and 2021 in Suva following peaks in COVID-19 cases and resulting restrictions in each country (table 1). The total number of trapping observations varied per survey when traps were not set in all 15 households or observations were excluded, as detailed below (table 1).

**Table 1.** Summary of mosquito trapping campaigns in 12 informal settlements in Makassar, Indonesia and 12 informal settlements in Suva, Fiji. Note that not all 15 households were sampled in each settlement and each survey round. Therefore, the total trapping observations per round varies.

	Date	Number of trapping observations	Number of settlements
Makassar, Indonesia	2018 October	120	12
	2019 January	145	12
	2019 April	143	12
	2019 July	142	12
	2019 October	135	12
	2020 January	136	12
	2021 December	72	10
	2022 January	134	11
	2022 August	126	11
	2023 January	134	11
	2023 July	128	11
	2024 January	119	11

Suva, Fiji	2019 August	153	12
	2019 November	134	12
	2020 February	106	12
	2020 July	92	12
	2020 October	110	12
	2021 January	101	12
	2022 February	113	12
	2022 July	89	12
	2023 February	87	12
	2023 August	100	12
	2024 January	131	12

Unbaited BG sentinel II (Biogents AG, Regensburg, Germany) traps were deployed in each house for three days, either indoors against a wall or in an undercover area outside the house, depending on the preference of the household and the availability of a power connection. Samples were collected and transported on ice to the laboratory where they were identified to sex and species under a dissection microscope according to morphological keys and counted (Rueda 1990, Reuben 1994). If a household member was not home at the time of trap deployment or declined to host a trap at that time, then a sample was not collected from that house during that survey round. Samples were excluded from analysis if the power to the trap was not running at the time of collection, indicating a loss of power during the sampling period. If a household member was not home during trap collection, the trap was collected at the next available opportunity and the number of deployment days was recorded.

### 2.2. Climatic, environmental and socioeconomic data

We characterised climatic, environmental and socioeconomic variation within and between settlements using data collected *in situ*, and open-source climatic and land cover data.

Household surveys to determine water use and supply were conducted in 617 households across the 12 informal settlements in Makassar between November 2018 and January 2019, and 792 households in Suva between June 2019 and August 2019 (Leder *et al* 2021). Respondents, preferentially the female head of household, were asked to describe their access to different water sources used for any purpose, including wells (shallow or deep), rainwater and piped water, and if water was stored from any of these sources. Settlements were classified as having piped water supply (more than 50% of households reported continuous access), intermittent piped water supply (more than 50% of households reported continuous or intermittent access) or no piped water supply (more than 50% of households reported no access). We estimated the relative extent to which households store water as the number of water sources stored, assuming that storing water from more sources equated to more water storage overall.

Subsequent quarterly surveys also collected information about the frequency and duration of flooding. Respondents were asked how many days in the previous three months the area outside or under their house was flooded. We computed the relative extent to which each settlement experienced flooding as the mean number of days flooding was reported, across all surveyed households, and all survey dates.

Local microclimate was measured *in situ* using iButton temperature and humidity loggers (Hygrochron DS1923; Maxim Integrated, San Jose, California). Five loggers were deployed outdoors in each settlement at approximately 1.5–2 m height in custom solar radiation shields, concurrent with the first round of mosquito trapping in each city (Ramsay *et al* 2021). Data were downloaded following mosquito trapping surveys, although logger loss, failure and fieldwork limitations mean that data were not retrieved for all loggers, or for all time periods.

To avoid excluding mosquito trapping observations without concurrent microclimate data, we derived continuous microclimate measurements over the entire study period, at the settlement level. We first calculated hourly settlement-level temperatures as the mean of all loggers in a settlement (excluding periods where data were retrieved for only one logger). We then developed Gaussian ordinary least squares linear regression models to predict settlement-level microclimate from temperature time series recorded at the nearest meteorological station (Erraguntla *et al* 2021). Meteorological data were sourced from the Integrated Surface Database (NOAA National Centers for Environmental Information 2001) for Station IDs 971800 (Makassar) and 916830 (Suva). Models were fitted separately for each settlement using the first six months of logger data (between 164 and 172 days for each settlement) and included the hour of measurement coded as a categorical variable as a predictor variable. The first six months of logger data represented the most complete time series of these data and included periods from both the wet and dry seasons in both countries.

Total daily precipitation measured at the meteorological station in each city was extracted from the National Centers for Environmental Information Global Surface Summary of the Day dataset (www.ncei.noaa.gov/access/search/data-search/global-summary-of-the-day; NOAA National Centers of Environmental Information 2024) To capture the effects of climatic variation across all stages of the mosquito lifecycle we summed total precipitation and computed the mean of daily mean, minimum and maximum predicted temperature over the 30 d prior to each trap deployment (Tantowijoyo *et al* 2016). To determine variation in land cover, we extracted the percentage of urban and vegetated pixels within each settlement using 10 m land cover maps from the European Space Agency WorldCover 2021 (v2; Zanaga *et al* 2021).

### 2.3. Causal modelling

We specified a directed acyclic graph (Arif and MacNeil 2022) to represent the putative causal relationships and interdependencies between climatic, environmental and socioeconomic variables, and mosquito abundance in urban informal settlements (figure 1), based on previous research (table S1). We tested the causal framework for the two most abundant mosquito vectors, *Ae. aegypti* and *Culex quinquefasciatus,* separately for each city and each mosquito species, using a structural causal modelling approach (Arif and MacNeil 2022).

To determine the casual effect of each predictor variable on mosquito abundance we selected covariates following the backdoor criterion so that all confounding pathways between the response and predictor variable of interest were blocked (figure 1; Laubach *et al* 2021; Arif and MacNeil 2022). For example, to estimate the causal effect of temperature on mosquito abundance, precipitation and land cover must be included as covariates to block their confounding effects (table 2; figure 1). Precipitation and land cover thus act as controls in the model and only the parameter estimate for the effect of temperature is interpreted. See Arif *et al* (2022) for a detailed example of applying the backdoor criterion to observational data. We fitted separate models for each predictor variable of interest, including the necessary adjustment covariates (table 1), but only present the effect estimates for the predictor variable of interest in figure 3 (all models, including adjustment covariates, are detailed in table 2). This approach avoids biased parameter estimates which are common when using information criterion (e.g. AIC) or stepwise selection to select predictor variables or infer causal relationships (Arif *et al* 2022, Arif and MacNeil 2022).

**Table 2.** Summary of causal models showing the adjustment covariates included for each predictor variable of interest. Note that the effect of water supply on mosquito abundance in Suva was not modelled as all 12 informal settlements had access to piped water supply (more than 50% households reported continuous access).

	Predictor variable of interest	Adjustment covariates
Makassar, Indonesia	Temperature	Precipitation, land cover
	Precipitation	Temperature, land cover, flooding, water storage
	Land cover	Temperature, precipitation, flooding
	Flooding	Precipitation, land cover
	Water supply	Precipitation, water storage
	Water storage	Precipitation

Suva, Fiji	Temperature	Precipitation, land cover
	Precipitation	Temperature, land cover, flooding, water storage
	Land cover	Temperature, precipitation, flooding
	Flooding	Precipitation, land cover
	Water storage	Precipitation

All models were fitted as generalised linear mixed models, assuming a negative binomial distribution, using the R package *glmmTMB* (Brooks *et al* 2017). The response variable was the total count (male and female) of *Cx. quinquefasciatus* or *Ae. aegypti* caught in each trapping observation (Makassar *n* = 1534, Suva *n* = 1216). All models included household, nested in settlement as a random effect to control for non-independence of repeated measures, an offset of the number of days a trap was deployed for to adjust for sampling effort, and the placement of a trap indoors or outdoors, coded as a categorical variable, to account for any systematic effects of trap placement (5% of traps were placed outdoors in Makassar and 8% in Suva). We selected between colinear variables (mean, minimum and maximum temperature; urban and vegetated land cover) based on the lowest AIC in univariate models, whilst keeping the selected variable consistent amongst species and locations for ease of comparison (tables S2 and S3). All continuous variables were scaled, by subtracting the mean and dividing by standard deviation, prior to model fitting. All models were checked for normality of residuals, over- or under-dispersion and zero-inflation using the R package *DHARMa* (Hartig 2022). We extracted model coefficients and their 95% confidence intervals using the *tidy* function in the R package *broom* (Robinson *et al* 2025) and calculated their exponents to derive incident rate ratios (IRRs). All data processing and analyses were performed in R (version 4.2.0; R Core Team 2021) on the Monash Secure eResearch Platform (Monash SeRP) to protect the privacy of settlement residents.

## 3. Results

Mosquito populations in informal settlements across Suva and Makassar were dominated by *Cx. quinquefasciatus* and *Ae. aegypti*. Across 12 sampling campaigns, including 1534 successful trap sets, 86 120 mosquitoes were caught and identified in Makassar (mean count 1.46 per trap per day). Across 11 sampling campaigns in Suva, totalling 1216 successful trap sets, 6134 mosquitoes were caught and identified (mean count 0.14 per trap per day). *Cx. quinquefasciatus* was the dominant species in Makassar, accounting for 92.9% of the total catch, followed by *Ae. aegypti* (6.4% of total catch). In Suva, *Cx. quinquefasciatus* was less dominant, but still the most abundant (58.8% of total catch), with *Ae. aegypti* making up 22.1% of the total catch. The counts of all species caught are detailed in table S4, although only *Cx. quinquefasciatus* and *Ae. aegypti* were sufficiently abundant to model. A small percentage of samples (4% and 8% in Makassar and Suva, respectively) were excluded from analysis (and not counted among successful trap sets) due to the trap being unpowered at the time of sample collection.

Our causal models showed that the magnitude and direction of relationships between mosquito abundance and climatic, environmental and socioeconomic drivers differed between informal settlement locations, and between vector species (figure 3; the raw results of all models are in table S5). We report IRRs for the effect of each predictor variable on mosquito abundance (figure 3). Here IRRs can be interpreted as the relative change in mosquito abundance for each predictor variable. An IRR of one means that there is no association between the predictor variable and abundance whilst an IRR above (below) one means that predictor caused higher (lower) abundance. For example, an IRR of 1.5 for the effect of piped water supply means that abundance is expected to be 50% higher in settlements with piped water supply compared to those without.

**Figure 2.**
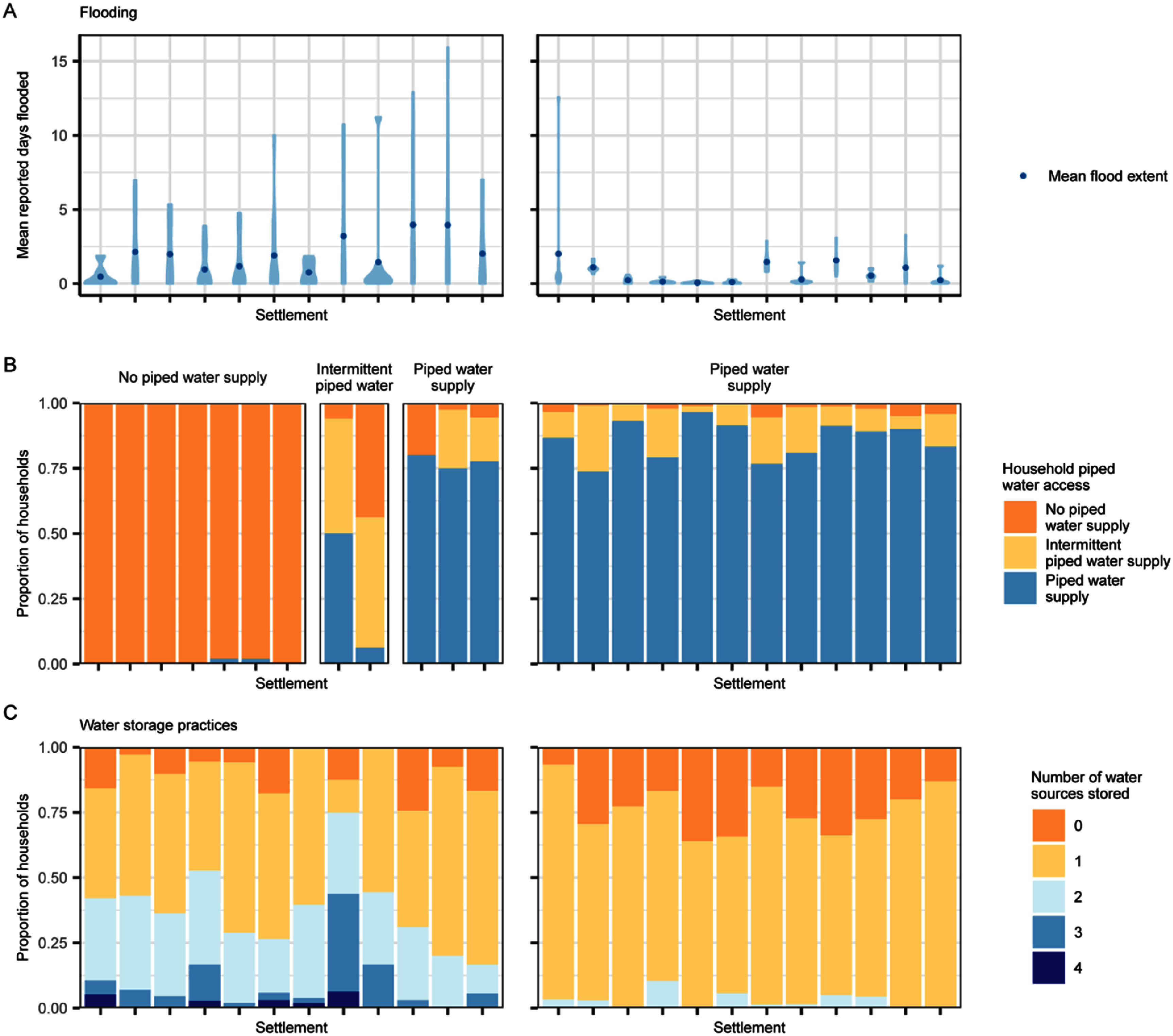
Flooding, water supply and water storage practices in informal settlements in Makassar, Indonesia and Suva, Fiji. (A) Mean days households reported flooding in the previous three months in each settlement and each survey round. Violin plots (shaded) represent the distribution of mean days flooded across all survey rounds. Points represent the mean days flooded across all surveys rounds in each settlement. (B) Household reported access to piped water supply. The plot grouping shows the water supply classification for each settlements whereas the colours show the proportion of households with or without piped water supply within each settlement. (C) Household reported water storage practices.

**Figure 3.**
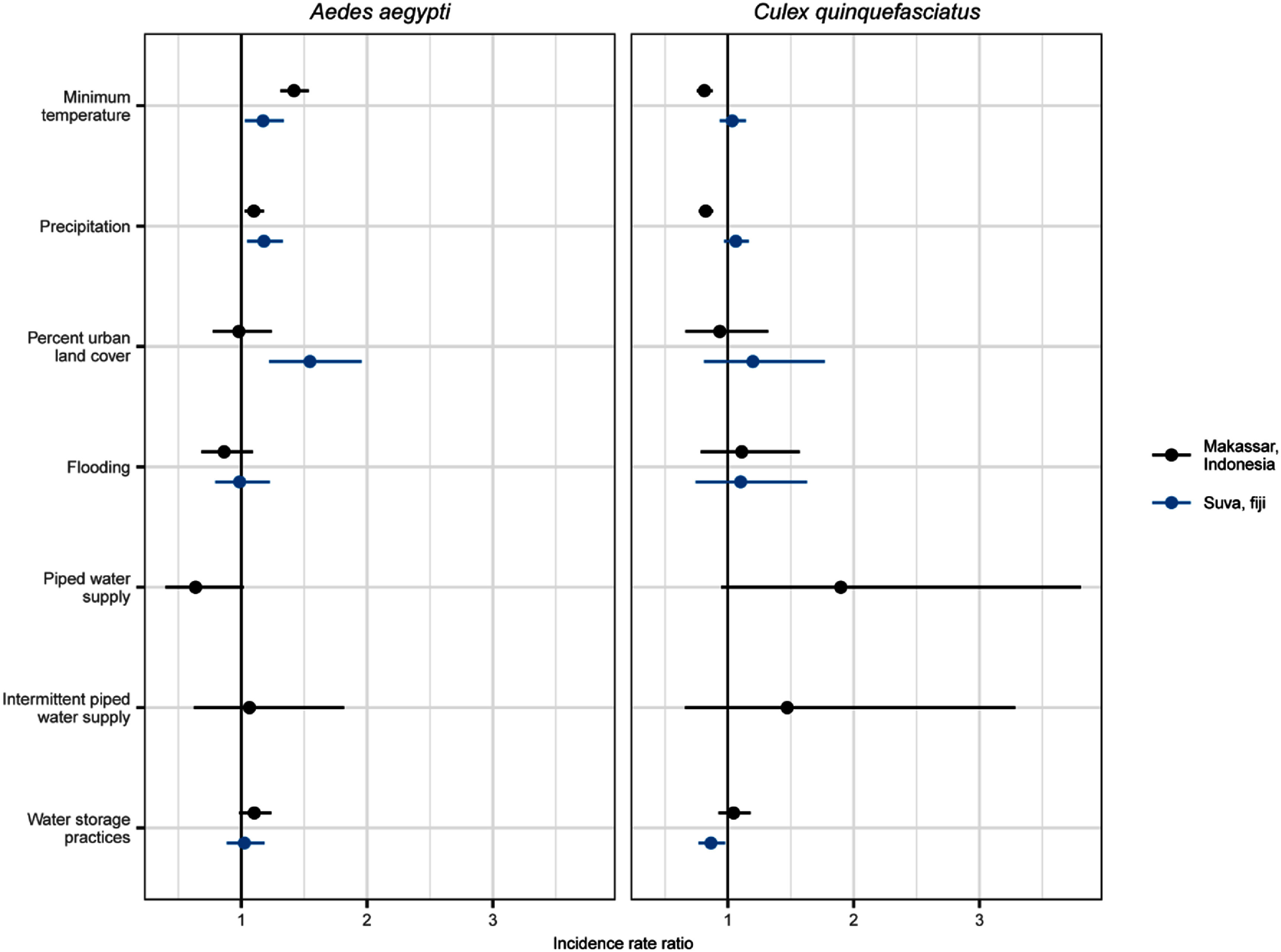
Climatic, environmental and socioeconomic drivers of *Aedes aegypti* and *Culex quinquefasciatus* in urban informal settlements in Makassar, Indonesia and Suva, Fiji. Points represent estimated incident rate ratios and lines 95% confidence intervals for the predictor variable of interest in causal models for each location and species. Significant effects of predictor variables can be determined when the 95% confidence intervals (lines) do not cross one.

Household-reported water access and storage practices varied within and between informal settlements (figure 2) and had contrasting effects on mosquito abundance (figure 3). Access to piped water supply, at the settlement level, had the largest effect, albeit with large uncertainty, on *Ae. aegypti* abundance in Makassar, where households within settlements with continuous access to piped water had considerably fewer *Ae. aegypti* (IRR 0.64 [0.40, 1.02]; figure 3). In contrast, intermittent access to piped water did not have the same protective effect of reducing *Ae. aegypti* abundance (IRR 1.07 [0.63, 1.82]; figure 3). Households in settlements with piped water supply also tended to have more *Cx. quinquefasciatus,* with a stronger positive effect in those with continuous access (IRR 1.90 [0.95, 3.81], figure 3).

All settlements in Suva had access to piped water supply (>74% households reported continuous access to piped water in all 12 settlements; figure 2(B)), although more than 50% of households in each settlement still reported storing water from at least one source (figure 2(C)). We could not, therefore, test the effect of piped water supply at the settlement-level in Suva. Households reporting more water storage practices in Suva had fewer *Cx. quinquefasciatus* (IRR 0.87 [0.77, 0.98]), but no effect on *Ae. aegypti* abundance was observed (IRR 1.03 [0.89, 1.19]; figure 3). Water storage practices had the opposite effect in Makassar, with no effect on *Cx. quinquefasciatus* abundance (IRR 1.04 [0.93, 1.18]) but a small positive effect on *Ae. aegypti* abundance (IRR 1.10 [0.98, 1.24]; figure 3).

The climatic drivers of *Ae. aegypti* were most consistent between geographic locations, with higher minimum temperature and higher precipitation driving higher abundance in both Makassar and Suva (figure 3). In contrast, the effects of temperature and precipitation on *Cx. quinquefasciatus* differed between cities, with lower temperatures and lower precipitation related to higher abundance in Makassar but with no relationship in Suva (figure 3). Over the study period (2018–2024), total annual precipitation averaged 2629 mm in Makassar and 2827 mm in Suva, whilst temperature averaged 27.7 °C in Makassar and 25.9 °C in Suva.

Urban land cover was only important for *Ae. aegypti* in Suva, where settlements with higher proportions of urban land cover (ranging from 6%–90% amongst the 12 informal settlements) had considerably more *Ae. aegypti* (IRR 1.55 [1.22, 1.96]). Meanwhile, the extent to which settlements experienced flooding had no effect on either mosquito species, or in either location, despite there being considerable variation in flood exposure amongst settlements (figures 2(B) and 3).

## 4. Discussion

Urban informal settlement populations are expected to grow to more than 3 billion people over the next 30 years (United Nations 2023). Given the concomitant growth in strategies to improve environments and livelihoods (Lilford *et al* 2017, Satterthwaite *et al* 2020, Leder *et al* 2021), much emphasis is being placed on understanding human health threats and their drivers in these settings (Lilford *et al* 2017, Hambrecht *et al* 2022). By investigating the causal drivers of mosquito abundance in two very different urban informal settlement locations we have identified the pathways through which changes in informal settlement environments are likely to alter vector dynamics, and the extent to which these pathways are generalisable for different species and in different geographic contexts.

Water supply, at the settlement level, was the strongest driver of mosquito abundance (albeit with large uncertainty, possibly reflecting the relatively small sample size of 12 settlements in each location), but with opposite effects on each vector. For *Ae. aegypti*, the primary dengue vector, continuous piped water supply reduced vector abundance in Makassar, in keeping with findings that piped water supply is protective against dengue (Schmidt *et al* 2011, Gibb *et al* 2023). In contrast, piped water supply also caused more abundant *Cx. quinquefasciatus*. Although not as prolific a vector as *Ae. aegypti, Cx. quinquefasciatus* is the primary vector of bancroftian filariasis and also a competent vector of Japanese encephalitis, West Nile virus and Chikungunya, amongst others (Bhattacharya and Basu 2016). Piped water supply generates a higher volume of grey water which, in Makassar, is discharged to open drains (Tjandraatmadja *et al* 2012), creating ample breeding habitat for *Cx. quinquefasciatus* which prefers to breed in polluted and nutrient-rich water (Bhattacharya and Basu 2016). Improvements in water supply and security, a priority in informal settlement upgrading programs (Olthuis *et al* 2015), could, therefore, inadvertently increase the risk of *Cx. quinquefasciatus-*borne viruses without sufficient mitigation (e.g. covering drains, preventing blockages from hard-waste).

The effects of water storage practices on mosquito abundance were much smaller and more ambiguous than those of piped water supply. Even in informal settlements with continuous piped water supply, including those in Suva with near-ubiquitous access, most households still reported storing water from at least one source, suggesting some level of water insecurity (e.g. cost, intermittent supply) regardless of access. The relatively small effects estimated for this driver may reflect the complexity of capturing the impact of household-scale variables within densely populated informal settlement environments. Alternatively, piped water supply may have other effects on mosquito abundance, independent of its impact on water storage practices. Although small, the estimated effects of water storage were consistent with that of water supply, where households storing water from more sources tended to have more *Ae. aegypti* in Makassar, and in Suva, increased water storage behaviour reduced the abundance of *Cx. quinquefasciatus* (likely indicating an impediment to piped water resulting in reduced grey water runoff).

Climatic variables were similarly important drivers of abundance, but with differential effects on each vector. For *Cx. quinquefasciatus*, high precipitation reduced abundance in Makassar, likely by flushing storm and grey water drains (Coalson *et al* 2021), but increased the abundance of *Ae. aegypti* in both locations. Evidence for increasing variability in precipitation due to global climate change (Zhang *et al* 2024) makes it especially difficult to project impacts on vector populations, especially when effects on vectors themselves are also variable (Franklinos *et al* 2019). Higher temperatures increased *Ae. aegypti* abundance across both informal settlement locations. Rising temperatures due to global climate change and growing urban heat islands are therefore likely to increase *Ae. aegypti* abundance, alongside exacerbating heat stress, which is already nearing the upper limits of human tolerance in some informal settlements (Ramsay *et al* 2021, 2023). Informal settlement upgrading programs may inadvertently worsen urban heat island effects by increasing the coverage of impervious surfaces and built structures, for example through mobility improvements (e.g. paved pathways and roads; Olthuis *et al* 2015) resulting in increased dengue risk (Wimberly *et al* 2020). Such effects can be mitigated by maintaining green spaces in informal settlements, for example, through nature-based solutions which concurrently deliver infrastructure such as improved sanitation (Satterthwaite *et al* 2020, Leder *et al* 2021).

Some effects were specific to each geographic location. Urban land cover was only important for *Ae. aegypti* in Suva where it was the strongest driver of abundance, likely through the addition of anthropogenic breeding habitat in more densely urbanised settlements (Franklinos *et al* 2019). Urban land cover in informal settlements in Suva was considerably lower than that in Makassar (mean 58.3% and 74.4%, respectively) suggesting that changes in urban land cover may only have an effect up to a certain level of urbanisation. We found no strong effects of flooding in either location or for either vector. As our household reported flood data was not temporally consistent with mosquito trapping we could not account for specific flood events, although previous research has found mixed evidence on the effect of flooding on mosquito-borne diseases, finding both increases, decreases and no effect (Coalson *et al* 2021). Moreover, our flood data did not account for different sources of flooding (e.g. tidal, pluvial or fluvial), thus did not distinguish between fresh and saline water, to which many mosquito species are sensitive. Future studies could specify more nuanced flooding variables, including timing, intensity and source, to accurately capture any effects on vectors.

There are some limitations in our methodological approach, especially pertaining to the complexities of fieldwork in dynamic environments such as urban informal settlements. We specified our climate, environmental and socioeconomic predictor variables at the household and settlement level and acknowledge that there are other factors that we did not capture (for this reason we included household nested within settlement as a random effect in our models). Household level factors could include the prevalence of electric fans and the porosity of houses (including windows and doors). Such variables are time-intensive to survey and their effects on mosquito abundance are difficult to estimate given the movement of mosquito populations between houses (Liew and Curtis 2004). Our socioeconomic and environmental variables relating to water supply and access, and flooding could be improved by including temporal components to capture their variation over time, concurrently with mosquito trapping. Power supply in informal settlements can be unreliable and intermittent (Satterthwaite *et al* 2020) and our mosquito traps required electricity to run. We excluded mosquito trapping samples when the trap was not powered at the time of collection. However, it is possible that power was lost during the three-day trapping period but switched on at the time of collection, meaning that the trap would not have been active for the complete three-day deployment. As our study was conducted over the COVID-19 pandemic there were disruptions to fieldwork resulting in some longer gaps between mosquito surveys, however the specification of our predictor variables was not affected by COVID-19 disruptions and therefore the impacts on the results of this study were minimal.

## 5. Conclusion

Here we clearly identify the pathways through which well-designed interventions can reduce the risk of mosquito-borne diseases in urban informal settlements. Improving water security is a priority in informal settlements which can concurrently reduce the burden of dengue by suppressing *Ae. aegypti* populations. Unintended effects on other vector populations including *Cx. quinquefasciatus* can be mitigated by improving grey water drainage systems where piped water is available and ensuring water storage containers (e.g. rainwater tanks) are covered. Managing the impacts of precipitation extremes and variability (i.e. both drought and heavy precipitation; Zhang *et al* 2024) will also be essential for water security as well as to mitigate the effects of precipitation on vectors (Lowe *et al* 2021). *Ae. aegypti* populations can be further reduced by mitigating urban heat islands, such as through maintaining green spaces and vegetation coverage, whilst also lessening exposure to heat stress (Jay *et al* 2021, Ramsay *et al* 2023). Nature-based solutions, which harness natural ecosystem services to deliver benefits to society (Lin *et al* 2021), have the potential to deliver all the above changes, with holistic benefits for human and environmental health. Leveraging this potential is essential to improve the livelihoods of the growing number of informal settlement residents and accelerate the implementation of Sustainable Development Goals 3 and 11.

## Data Availability

The data cannot be made publicly available upon publication because they contain sensitive personal information. The data that support the findings of this study are available upon reasonable request from the authors.
